# Evidence for low high-density lipoprotein cholesterol levels in Australian indigenous peoples: a systematic review

**DOI:** 10.1186/1471-2458-14-545

**Published:** 2014-06-02

**Authors:** Jasmine G Lyons, Kerin O’Dea, Karen Z Walker

**Affiliations:** 1 Baker IDI Heart and Diabetes Institute, 75 Commercial Road, Melbourne, Victoria 3004, Australia; 2 Department of Medicine (St. Vincent’s), University of Melbourne, Clinical Sciences Building, 29 Regent Street, Fitzroy, Melbourne, Victoria 3065, Australia; 3 School of Population and Global Health, University of Melbourne, 207 Bouverie Street, Melbourne, Victoria 3065, Australia; 4 School of Population Health, Division of Health Sciences, University of South Australia, North Terrace, Adelaide, South Australia 5000, Australia; 5 Department of Nutrition and Dietetics, Monash University, 264 Ferntree Gully Road Notting Hill, Victoria 3168 Melbourne, Australia

**Keywords:** Indigenous, Australian Aborigines, Torres Strait Islanders, Lipids, Dyslipidaemia, High-density lipoprotein cholesterol, Cardiovascular disease, Cardiovascular disease risk factors, Cardiometabolic, Non-communicable disease

## Abstract

**Background:**

Low plasma high-density lipoprotein cholesterol (HDL-C) levels are a strong, independent, but poorly understood risk factor for cardiovascular disease (CVD). Although this atherogenic lipid abnormality has been widely reported in Australia’s Indigenous peoples, Aboriginal and Torres Strait Islanders, the evidence has not come under systematic review. This review therefore examines published data for Indigenous Australians reporting 1) mean HDL-C levels for both sexes and 2) factors associated with low HDL-C.

**Methods:**

PubMed, Medline and Informit ATSI Health databases were systematically searched between 1950 and 2012 for studies on Indigenous Australians reporting mean HDL-C levels in both sexes. Retrieved studies were evaluated by standard criteria. Low HDL-C was defined as: <1.0 mmol/L. Analyses of primary data associating measures of HDL-C with other CVD risk factors were also performed.

**Results:**

Fifteen of 93 retrieved studies were identified for inclusion. These provided 58 mean HDL-C levels; 29 for each sex, most obtained in rural/regional (20%) or remote settings (60%) and including 51–1641 participants. For Australian Aborigines, mean HDL-C values ranged between 0.81-1.50 mmol/L in females and 0.76-1.60 mmol/L in males. Two of 15 studies reported HDL-C levels for Torres Strait Islander populations, mean HDL-C: 1.00 or 1.11 mmol/L for females and 1.01 or 1.13 mmol/L for males. Low HDL-C was observed only in rural/regional and remote settings - not in national or urban studies (n = 3) in either gender. Diabetes prevalence, mean/median waist-to-hip ratio and circulating C-reactive protein levels were negatively associated with HDL-C levels (all P < 0.05). Thirty-four per cent of studies reported lower mean HDL-C levels in females than in males.

**Conclusions:**

Very low mean HDL-C levels are common in Australian Indigenous populations living in rural and remote communities. Inverse associations between HDL-C and central obesity, diabetes prevalence and inflammatory markers suggest a particularly adverse CVD risk factor profile. An absence of sex dichotomy in HDL-C levels warrants further investigation.

## Background

Preventable chronic disease, including cardiovascular disease (CVD) and type 2 diabetes, accounts for 70% of the difference of burden of disease between Indigenous and broader Australian populations, and is now regarded as one of the greatest causes of current inequities in Indigenous health status [1]. CVD risk factors have been consistently shown to be more pronounced in Indigenous populations and include much higher rates of type 2 diabetes, cigarette smoking, overweight/obesity, alcohol misuse, poor nutrition, depression [2], and the Metabolic Syndrome (MetS), a cluster of various CVD and diabetes risk factors [3,4]. Alongside this burden of preventable non-communicable disease, high rates of infectious disease represent an important cause of morbidity and mortality in Indigenous Australians compared to the broader population [5-10].

One critical aspect of this adverse CVD risk factor profile is the high prevalence of low high-density lipoprotein cholesterol (HDL-C). A low HDL-C level is strongly and inversely associated with risk for coronary heart disease [11]. High HDL-C exerts its cardio-protective effect primarily through its role in reverse cholesterol transport and its anti-inflammatory, anti-thrombotic and anti-oxidative properties. As endogenous oestrogens have a favorable effect on lipid metabolism, females often have higher HDL-C levels than males [12]. Also, alcohol consumption can increase HDL-C levels. Various metabolic and environmental factors, including the presence of acute or chronic infection, a high intake of refined carbohydrate and cigarette smoking, can decrease HDL-C levels. Importantly, low HDL-C is likely to be a secondary effect from components of the MetS: insulin resistance, central obesity, high triglyceride dyslipidaemia and chronic, low-grade inflammation [13]. To date, no specific clinical targets for HDL-C levels have been determined; thus low HDL-C has been variously defined [14-19]. In Australia, lipid management guidelines from the National Heart Foundation and the Cardiac Society of Australia and New Zealand have set a threshold for low HDL as < 1.0 mmol/L [17].

Multiple reports describe extremely low HDL-C levels in Australian Indigenous populations [8,9,20-25]. In 1979 very low mean HDL-C levels (0.85 mmol/L) were reported from a small, rural- Western Australian cohort of young Aboriginal men (<35 years of age) [26] and from a rural community in central Australia, where mean HDL-C levels decreased over an eight year period as obesity and diabetes prevalence increased [22]. Using the same criteria, a recent report determined low HDL-C prevalence in a national Indigenous cohort as 70.2% [27]. Moreover, low HDL-C is not confined to adults: by the National Cholesterol Education Program Adult Treatment Panel III (NCEP ATP-III) definition of < 40 mg/dL (<1.04 mmol/L), low HDL-C was evident in 30% of Indigenous children/adolescents (9–14 years) from Northern Australia [28]. Crucially, in a 2004 study of 913 participants from Aboriginal communities in central and northern Australia, 95% of women and 89% of men were found with low HDL-C (<1.0 mmol/L) a problem apparent even among those individuals who were lean and without other abnormalities indicative of MetS [29].

Given the consistency in reports of low HDL-C in Australian Indigenous people, we sought to establish whether these findings could be generalized across populations and settings. Our aims were to: 1) collate and summarize published data reporting mean HDL-C levels for both females and males; and 2) where possible, analyze associations between low HDL-C with other CVD clinical risk factors.

## Methods

### Definitions

We use the term ‘Indigenous Australians’ to refer to individuals identifying as ‘Aboriginal’ or ‘Torres Strait Islander’. Study locations (e.g. remote, urban) were defined as described within each study.

### Search strategy

Using a multi-field search we conducted a search in the PubMed, MEDLINE (Web of Knowledge), and Informit ATSI Health databases. Search terms were:

cholesterol/high density lipoprotein/HDL-C/lipoprotein/dyslipid*/lipid;

Aborigin*/Indigenous/Torres Strait Islander/Oceanic ancestry group;

cardiovascular disease/heart disease/coronary heart disease;

metabolic syndrome/MetS;

Australia.

Truncation of terms captured any variation in terminology. Studies retrieved were restricted to full-article, English-language publications published between January 1950 and January 2012. Additional data sources were obtained by hand search. Narrative reviews, editorials, letters, commentaries and grey literature (to ensure inclusion of only reliable, peer-reviewed data) were excluded. Two investigators (JGL and KZW) conducted the literature searches independently and results were combined.

### Study inclusion criteria

Published original quantitative studies were included for review if they fulfilled the following selection criteria:

1. The study was based solely on Australian Indigenous populations, or provided a separate analysis of Indigenous participants

2. Standard values (mean, median or geometric mean) of serum or plasma HDL-C levels were reported separately for males and females.

As there is no single standard for low HDL-C, the presentation of mean HDL-C values rather than prevalence rates for low HDL-C were used as an inclusion criterion. We defined low HDL-C as <1.00 mmol/L, as per Australian clinical guidelines [17]. For all included studies, we required presentation of both male and female values for HDL-C in order to examine gender differences and calculate the female:male HDL-C ratio [12].

### Data extraction/study quality assessment

Summary data for included studies were extracted into a standardized tool that included: citation, study population, year(s) of study, region (national, urban, rural/regional or remote), mean sample age/age range, sample size, response rate, mean HDL-C levels, and method of HDL-C measurement. Where possible, the following outcomes were also extracted as mean values: sex-specific anthropometric measurements (body mass index [BMI], waist circumference [WC], waist-to-hip ratio [WHR]), age, plasma C-reactive protein (CRP)) plus prevalence rates for diabetes, obesity, current tobacco smoking or infectious disease states. If multiple reports were available for a given study, the most recently published report or the report that provided more comprehensive results was selected for review. The quality of each study was assessed according to study-specific criteria (Table 1), based on previous general recommendations [30]. Each criterion contributed two points to an overall score of 10. A score of 10 points was considered very high quality; 8–9 high quality; 7–6 moderate quality; and < 5 low quality. Different surveys reported in a single study [22] were assessed separately. HDL-C data from the 2000 AusDiab study [31] - a national randomized cluster study- provided the non-Indigenous, Australian population reference. To examine possible associations, mean values of various CVD risk factors obtained from study results were plotted as independent variables against mean HDL-C levels, with each sex-specific value aggregated for each analysis between HDL-C and other variables: mean BMI, WC, WHR, age, CRP and prevalence of current smoking and obesity. The degree of association was assessed by Pearson’s correlation.

**Table 1:** Study quality assessment tool

**Study quality criteria**	**Example of high score**
Selection criteria used	Appropriate inclusion/exclusion criteria; low response rates kept to a minimum or possible explanations provided
Measurement of study variables	Validated method for lipid measurement; patient self-reports validated against appropriate existing records
Design specific sources of bias	Identification and selection of participants limited (e.g. only diabetic patients)
Use of appropriate statistics	Primary analysis of effect and control of confounding in analyses
Possible sources of bias	Statement of funding sources, conflict of interest

Legend:

Each criterion was assessed as either high or low score.

## Results

**Figure 1:**
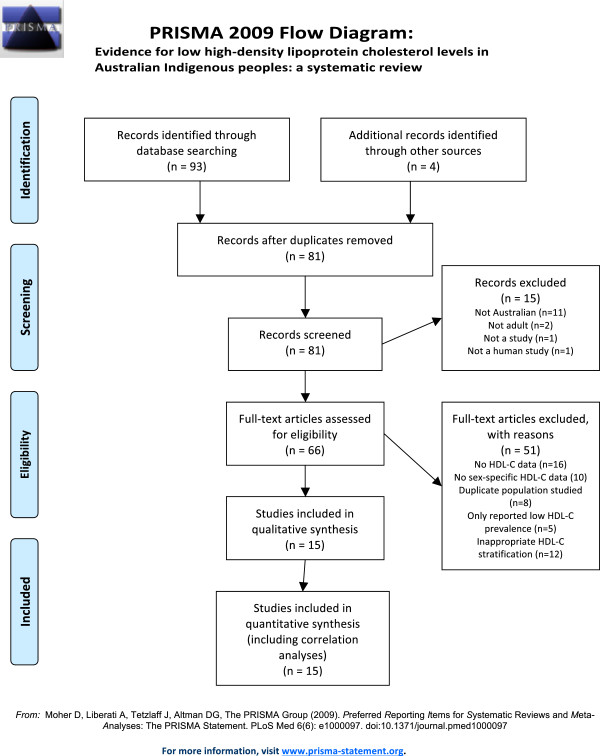
Literature search strategy (PRISMA flowchart).

Overall 93 articles were obtained in the initial literature search while another four were retrieved by hand-search. After evaluation, 15 studies were included for further qualitative and quantitative analysis (Figure 1). Their review was undertaken according to PRISMA guidelines for systematic reviews (see checklist, Additional file 1).

Most included studies were of either high (41%) or moderate quality (59%)- none were of very high or of low quality. All were cross-sectional assessments taken at a single time point, except one [22], where three sequential cross-sectional surveys were undertaken across a single community (1987, 1991 and 1995). Here, because retention rates varied across sex and age group, and cases were not matched across time points; data from each year were extracted and presented separately.

Sample sizes varied in size and location (Table 2), from n = 51, (the adult population in a remote community in Western Australia [9]) to n = 1641 (23 communities in far north Queensland [32]). Most studies had a strong regional focus; 8 of 15 were conducted in single rural or remote communities in the Northern Territory. One national survey included participants from medical practices in capital cities and from remote communities [33] and two urban surveys were carried out in metropolitan capital cities [34,35]. Six of the 15 studies presented HDL-C data in separate groups according to age, but where a mean overall age was reported, this ranged only between 29.9-40 years, for both males and females. While one study included certain paediatric data [9], data examined here concern only those aged over 15 years.

**Table 2:** Summarized findings of low high-density lipoprotein cholesterol in reviewed studies, grouped by population

**Reference**	**Year(s) study conducted**	**Population, location**^**1**^	**State/territory**	**Low mean**	
				**HDL-C**^**2**^	
				**Male**	**Female**
Indigenous populations					
[33]	2007-08	National survey^3^	NSW, QLD, Central Australia	No	No
[35]	2003-05	Urban survey from one metropolis^4^	NT	No^6^	No
Australian Aboriginal populations					
[37]	1986	1 remote community	NT	No	No
[38]	1987	1 remote community	NT	No	No
[39]	1987-88	1 regional community	VIC	No	No
[9]	≈ 1995	1 remote community, Great Sandy Desert	WA	Both genders	
[23]	1988	1 remote community, homeland residences	NT	Both genders^6^	
[22]	1987	1 rural community	NT	No	No
	1991			Both genders^7^	
	1995			Both genders^7^	
[21]	1996	1 remote community	NT	Both genders	
[8]	1999-2000	1 remote community, East Arnhem Land	NT	No	Yes
[36]	1992-95	1 remote community, Tiwi Islands	NT	No	No
[20]	2001-03	1 remote community, East Arnhem Land	NT	No	Yes
[34]	2000	Urban study, one metropolis	WA	No	No
[24]	1993-95	11 remote communities,	NT, far-north QLD	No^7^	No
[32]	1999-2000	23 rural communities	Far-north QLD	No^7^	No
Torres Strait Islander populations					
[24]	1993-95	11 remote communities	NT, far-north QLD	No^7^	No
[32]	1999-2000	23 rural communities	Far-north QLD	No^7^	No

Legend:

^1^Ethnicity is reported as defined in the original study.

^2^Low HDL-C defined as mean HDL-C value < 1.00 mmol/L.

^3^90%AA, 9% TSI, 1% both AA and TSI.

^4^84% AA; 6% TSI; 11% both.

^6^HDL-C levels stratified by age: 15–34 yrs and >35 yrs of age. In both age groups, results were the same.

^7^HDL-C levels stratified by age into: 15–24 yrs, 25–34 yrs and >35 yrs of age. For all age groups, results are the same.

*Abbreviations:HDL-C* high-density lipoprotein cholesterol, *AA* Aboriginal ethnicity, *TSI* Torres Strait Islander ethnicity, *NSW* New South Wales, *QLD* Queensland, *NT* Northern Territory, *VIC* Victoria, *WA* Western Australia.

Women were often overrepresented. Only three studies had fewer females than males: 39% [9]; 48% [36]; and 49% [32], while in all other studies the proportion of females ranged from 54-86%. Although some study response rates were low (down to 14%) [34,35], six of 15 studies reported response rates ≥ 80% [9,23,33,37-39] and one study reported response rates of between 57-97%, depending on survey year [22]. Two studies did not report a response rate [20,24] and in another, data were retrieved from medical records so that a community response rate was not applicable [33]. Another study reported male and female response rates separately; these varied from 53% (males, 1995) to 96% (females, 1989) [22]. Included studies were conducted between 1987-2008.

**Figure 2:**
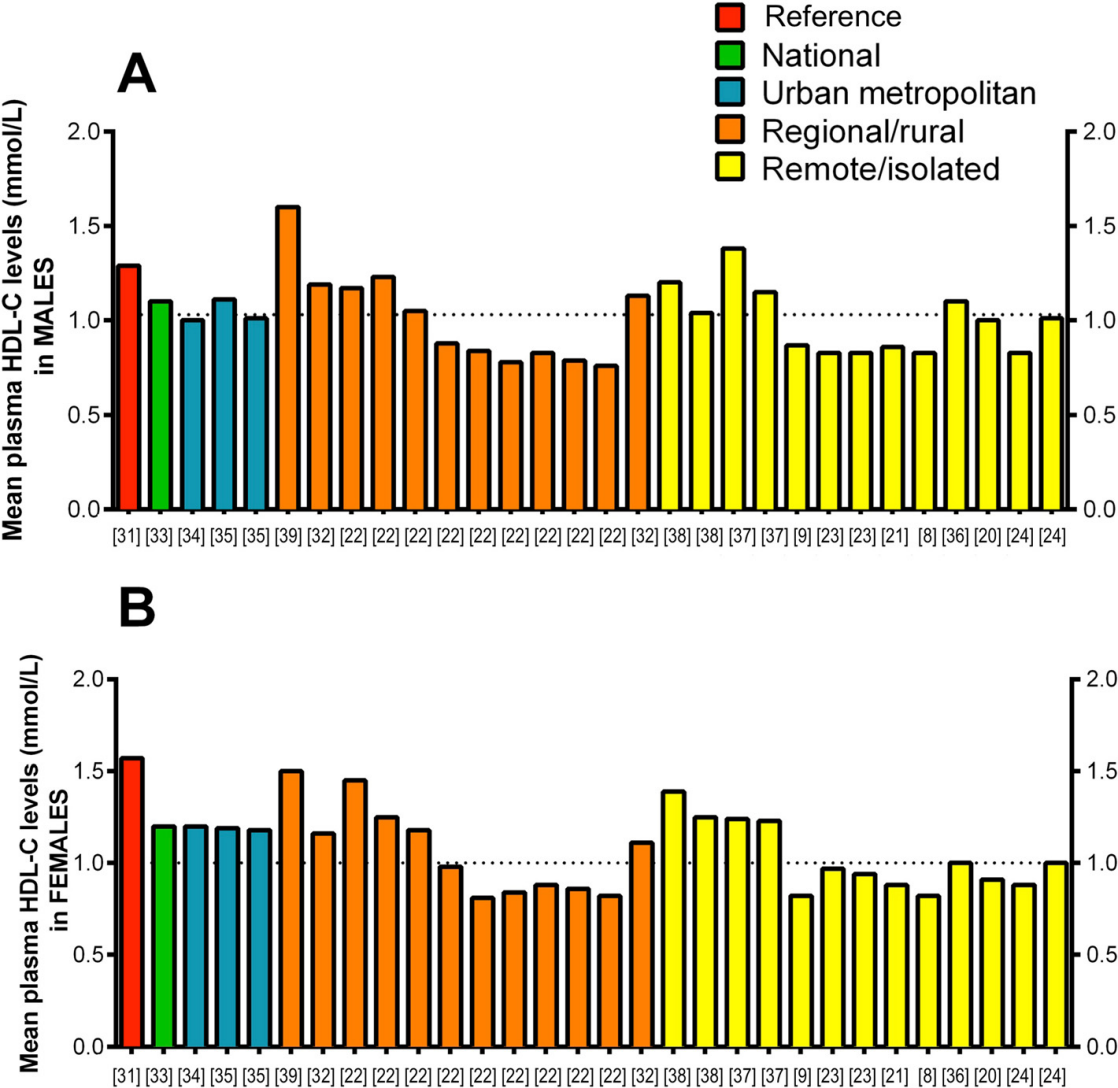
**Mean high-density lipoprotein cholesterol (HDL) levels in [A] males and [B] females, according to region.** The reference value was taken from a national survey of non-Indigenous Australians [31]. Each bar represents a single measurement; some studies have reported ≥2 values owing to stratification by ethnicity [24,32], age group [22,23,35,37,38] or year of survey [22].

In total, included studies reported 58 sets of HDL-C observations (29 for each gender). Mean HDL-C varied across studies (Figure 2: male [A] and female [B]). The lowest mean HDL-C reported was from a rural community in the Northern Territory, where mean HDL-C was 0.76 mmol/L in males aged < 35 years (1991 survey) and 0.81 mmol/L in females aged 15–24 years (1995 survey) [22]. This contrasts with results from a 1987–88 study in regional Victoria, where the highest mean HDL-C levels were reported: 1.6 mmol/L in men and 1.5 mmol/L in women [39]. Indeed, only two studies noted such high mean HDL-C values for men exceeding the mean value from non-Indigenous reference populations of 1.27 mmol/L [31]: 1.60 mmol/L [39] or 1.38 mmol/L [37]. In females, no other studies found mean HDL-C values above the non-Indigenous reference (1.57 mmol/L [31]). Eleven of fifteen studies did not report whether gender differences in HDL-C levels were of significance. Of the four that made this analysis, two studies found no significant difference [8,37] and one reported significantly lower HDL-C levels in females [20]. The fourth study found Aboriginal women to have significantly higher HDL-C than Aboriginal males, although no difference in HDL-C between genders was found among Torres Strait Islanders [24].

While there were no clear gradients in HDL-C according to region, Figure 2 shows that low levels of HDL-C generally predominated in rural and remote-dwelling Indigenous populations. This pattern is confirmed in collated study data (Table 2): all studies reporting low HDL-C in both males and females were conducted in remote or rural areas. This contrasts with the national AusDiab cohort, in which mean HDL-C was not low in either gender. Data from urban metropolitan areas were also consistent; neither the study from Darwin, Northern Territory [35], nor that from Perth, Western Australia [34], showed low mean HDL-C for males or females. Both these studies however, were subject to low response rates: 14% and 5-20% (depending on age group), respectively. Only two studies reported HDL-C values specifically for Torres Strait Islander populations, and neither showed low mean HDL-C [24,32].

Laboratory methods of HDL-C measurement are detailed in Additional file 2. The majority of studies report standardized, full or partially automated techniques, whereas HDL-C isolation or precipitation was carried out in only four studies. One study did not state method of HDL-C measurement [36], and in another, varied methods were likely, subject to the practice at each medical service from which medical record data were obtained [33].

**Figure 3:**
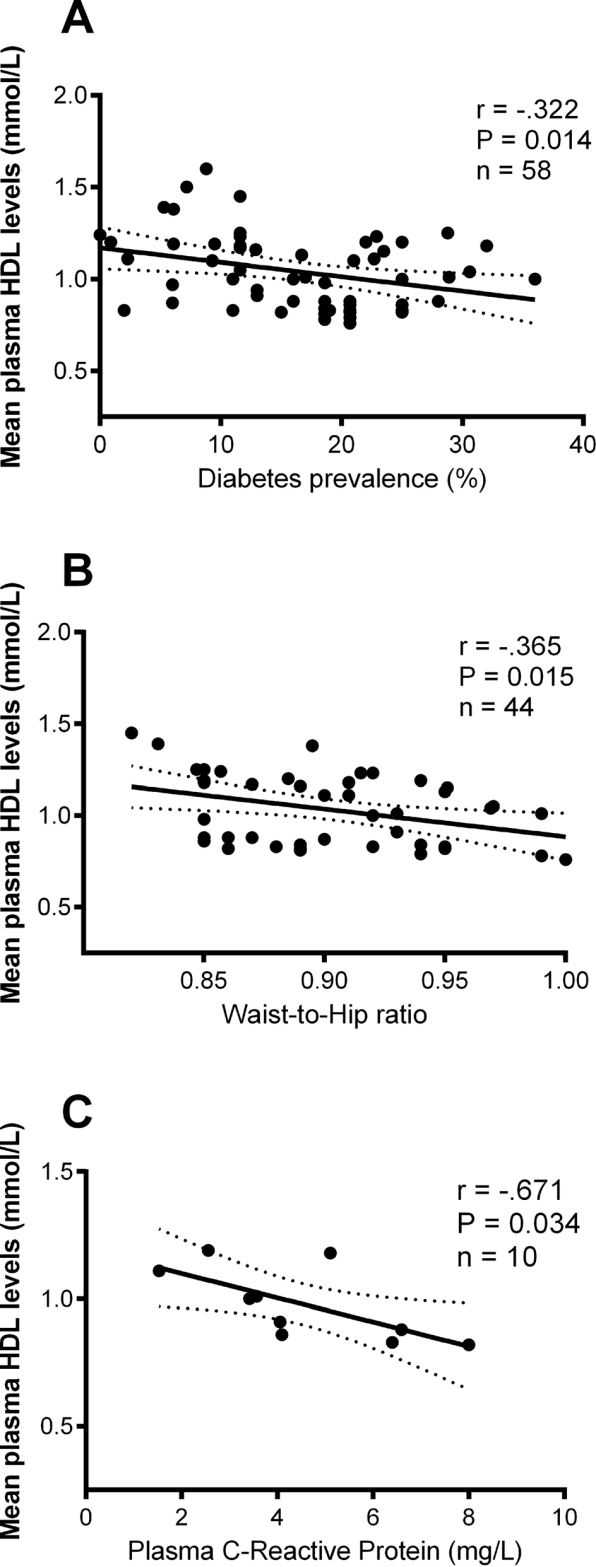
Relationships between mean high-density lipoprotein (HDL) cholesterol levels and selected cardiovascular risk factors: Diabetes prevalence (%) [A]; Waist-to-hip ratio [B]; Plasma C-Reactive Protein (mg/dL) [C].

Female:male HDL-C ratios are described in Additional file 2. One third of measurements (10 of 29 pairs in total) reported lower mean HDL-C levels in females than in males (i.e. ratio < 1.0), which contrasts with the ratio of 1.27 seen in the non-Indigenous reference population [31]. Overall, the lowest female:male ratios observed were in rural/regional and remote settings.Using data available from included studies, mean HDL-C values were negatively associated with prevalence of diabetes, mean WHR and mean/median plasma levels of CRP (Figure 3). Effect modification by gender was also observed for each of these variables. Diabetes prevalence was significantly inversely associated with HDL-C levels in males (r = −.373, P = 0.046) but not females (r = −.314, P = 0.1). Similarly, CRP levels were significantly associated with HDL-C levels in males (r = −.910, P = 0.03), but not females (r = −.728, P = 0.16) whereas WHR was significantly associated with HDL-C in females (r = −.428, P = 0.047) but not males (r = −.288, P = 0.19). Mean BMI, WC, age and prevalence of current smoking and obesity did not provide evidence of significant relationships with HDL-C levels in either sex-specific or combined correlation analyses (all P > 0.4).

## Discussion

Our analysis presents collated evidence of low HDL-C as a striking CVD risk factor in Aboriginal and Torres Strait Islander populations. While Indigenous Australians do not constitute a homogeneous group with respect to low HDL-C dyslipidemia, very low HDL-C appears particularly prevalent in rural and remote communities.

Diabetes prevalence is high in Indigenous populations [40]. Thus co-morbidities of diabetes and the MetS are likely to be strongly contributing, if not directly causal, factors in the presentation of low HDL-C. In reports from which we could source data, we found negative associations between mean HDL-C levels and diabetes prevalence, WHR and CRP levels, all well-recognised CVD risk factors in Indigenous communities [8,41-44]. In remote communities, where we found the lowest HDL-C levels, poor nutrition, excessive smoking and a sedentary lifestyle have been well documented. In turn, these factors are known to contribute to elevated plasma levels of the inflammatory marker CRP [45], which we found negatively associated with HDL-C. Although other risk factors known to be prevalent in Aboriginal populations such as smoking and renal disease [2] were prominent in our included studies they failed to impact on HDL-C in our pooled analyses.

Some populations in rural/regional and remote/isolated locations did not exhibit low HDL-C levels [32,37,38]. Two factors: absence of obesity and high alcohol intake may explain this. One of these studies came from an isolated non-obese community, where mean BMIs were 20.4-23.5 kg/m^2^[37]. Similarly, whereas an initial cross-sectional study from a rural community in the Northern Territory, reported high mean HDL-C levels, a subsequent survey 8 years later, indicated both significant weight gain, and a reduction in HDL-C levels [22]. Secondly, alcohol consumption may be critical; a study conducted in a small isolated community in the Northern Territory, found that ‘drinking’ (defined as those who consumed any alcohol) reduced the risk of low HDL-C (odds ratio: 0.4, 95% CI 0.2-0.6) [46]. The contribution of alcohol consumption to higher HDL-C in some populations is also suggested by some studies in remote locations [37-39]. In addition in one large study across 23 rural communities, where higher HDL-C levels were evident the prevalence of ‘drinking’ ranged from 51%-82% [32]. Also, Guest *et al.* acknowledge that alcohol consumption may have influenced the high HDL-C levels seen in another regional population [39].

The preponderance of studies undertaken in rural/remote settings was a prominent finding, typical of broader Indigenous health research [47]. Owing to the sampling method of AusDiab, which excluded districts if they were classified 100% rural [48], we could not directly compare HDL-C levels between Indigenous and non-Indigenous populations by location. However, in a population survey conducted wholly in non-indigenous rural populations in south-eastern Australia, mean HDL-C levels reported were 1.59 mmol/L for women and 1.33 mmol/L for men [49]. These levels are comparable to the AusDiab cohort, and much higher than in Indigenous populations from rural areas included in this review.

Low HDL-C dyslipidaemia clearly results from a complex interplay of environmental and genetic factors [50] with heritability estimated at 40-80% [51]. Indeed, the Turkish Heart Study found that high prevalence of low HDL-C (70% of men, 50% of women) could be attributed to a 25-30% higher hepatic lipase activity, suggesting a strong genetic component [52]. Few data however are available to link genetic predisposition to low HDL-C levels in Australian Indigenous populations. One early study found that 26% of a small population from the remote Kimberley region of northwestern Australia carried the ϵ4 allele for the APOE gene, known to be associated with raised plasma cholesterol and higher risk of CVD [53]. Furthermore, in a medium-sized study (n = 155) in southeast Queensland, APOE4 polymorphisms were 1.8 times more prevalent in Indigenous than non-Indigenous participants and associated with high triglycerides and low HDL-C [54]. Thus genetic differences cannot be unequivocally excluded as possible contributors to differing HDL-C phenotype across our study settings, while genetic admixture may contribute to the higher HDL-C observed in urban areas [34,35]. One exception here is a small, early study from northern Western Australia, where urban Aboriginal men had higher HDL-C levels than rural men. As both groups were identified as ‘full-blood Aboriginal’, factors such as urbanization and higher alcohol consumption may have been critical modifiers of lipid levels [26]. Early life epigenetic modifications may also potentially impact on HDL-C levels [55]. Nevertheless, the body of evidence is clear: non-genetic and modifiable influences appear critically important in the development of low HDL-C dyslipidemia.

Although studies in predominantly North American and western European populations indicate that women have higher HDL-C levels than men, data collated here from rural and remote Indigenous communities indicate this is not necessarily always the case. Different populations display different magnitudes in these sex differences [56]. Other populations of impoverished people (including rural poor in Andhra Pradesh, southern India [57]; Indigenous tribes in Himalayan India [58]), and Indigenous populations undergoing rapid epidemiologic transition in northern Russia, show similar low HDL-C levels in both women and men [59].

As a clinical consequence of a high burden of disease, low HDL-C in the Australian Indigenous population appears intrinsically linked to adverse socio-demographic determinants driving the high rate of poor health (e.g.: education, occupation and income) [60]. Both acute infection and chronic inflammation reduce HDL-C levels [13]. Infection/chronic inflammation are common in Indigenous Australians, particularly those from remote areas, where parasitic, fungal, viral and bacterial infections are promoted by poor household infrastructure, overcrowding and overdue vaccinations [61]. Accordingly, high CRP levels are a common CVD risk factor [41,44]. Communicable disease appears to be a potentially important contributor to the highly prevalent low HDL-C and high CRP reported here as evidenced by the negative association we found between mean CRP and mean HDL-C levels. This is consistent with other data [8,21] in Indigenous populations, including inverse associations seen between HDL-C levels and other atherogenic biomarkers such as vascular cell adhesion molecules [21] and fibrinogen [8,24]. One small, prospective epidemiological study of critical care patients in central Australia found that despite similar low HDL-C levels upon admission, HDL-C levels only remained low six months after discharge in the Indigenous group (median HDL-C: 0.8 mmol/L vs. 1.5 mmol/L in non-indigenous patients) [62]. Also, erythrocyte sedimentation rate, a marker of chronic inflammation, was significantly higher in the Indigenous patients at follow-up. Significantly, pro-atherogenic changes to HDL-C during states of infection/inflammation may accelerate CVD risk, beyond the risk conveyed by low HDL-C *per se*[13].

Our study has some limitations. Due to the small number of often cluster-based studies, external validity with respect to the wider or national Indigenous population in Australia must be uncertain. Nevertheless, we are confident that we give an accurate representation of low HDL-C prevalence in rural and remote communities. One potential source of bias is the variety of methods used to determine HDL-C especially in earlier studies (1980s-90s), although over the last 15 years methodology has been largely consistent. Studies providing only percentage prevalence of low HDL-C at different thresholds were excluded. Although their inclusion would expand the evidence base, it does not aid in finding an estimate of the prevalence of low HDL-C.

Associations about the impact of traditional CVD risk/‘lifestyle’ factors on the presentation of low HDL-C are now well recognized. However, research of lipid-specific metabolic dysfunction is likely to provide further insight into the extent it contributes to CVD risk in such high-burden populations. O’Neal *et al*. [25] identified a particularly atherogenic lipid profile in Aboriginal communities across northern Australia, that was characterized not only by low HDL-C, but also by an increase in small, dense LDL particles known to be highly susceptible to oxidative modification. Dysfunctional changes to lipoprotein composition may also be occurring for HDL as for LDL particles. For example, examination of the role of apolipoprotein-A1, which governs the ability of HDL to perform reverse cholesterol transport, would be valuable in assessing the relative importance of particle number, HDL-C content and function on CVD risk in Australian Indigenous populations. Examination of relationships between HDL-C and infectious disease load in these populations would also be valuable. If confirmed, these features together represent a very high-risk cardio-metabolic profile and may go some part in explaining the excess CVD morbidity and mortality that exists in Indigenous populations.

## Conclusions

This review confirms that low HDL-C levels are common in Australia’s Indigenous populations, and predominate in rural and remote locations. Associations found between HDL-C levels and other CVD risk factors that are also widespread in these communities provide aggregated evidence linking HDL-C and other features of cardio-metabolic risk, including a relationship between HDL-C and the inflammatory marker CRP. Such interrelationships provide a compelling case in support of comprehensive preventive health programs to reduce the burden of both communicable and non-communicable disease in Indigenous populations.

## Abbreviations

HDL-C: High density lipoprotein cholesterol; CVD: Cardiovascular disease; CHD: Coronary heart disease; AA: Australian Aborigine; BMI: Body mass index; F: Female; M: Male; MetS: Metabolic syndrome; TSI: Torres Strait Islander; WC: Waist circumference; WHR: Waist-to-hip ratio; CRP: Plasma C-reactive protein; DM: Diabetes mellitus; IGT: Impaired glucose tolerance; PEG: Polyethylene glycol precipitation; CS: Current smokers; OB: Obesity; GM: Geometric mean; NSW: New South Wales; QLD: Queensland; NT: Northern territory; VIC: Victoria; WA: Western Australia.

## Competing interests

The authors declare no competing interests.

## Authors’ contributions

KZW conceived of and designed the review. JGL and KZW completed literature search and extracted the data. JGL drafted the manuscript with input from all authors. KZW and KOD revised manuscript critically for important intellectual content. All authors contributed to analysis and interpretation of data and read and approved the final manuscript.

## Pre-publication history

The pre-publication history for this paper can be accessed here:

http://www.biomedcentral.com/1471-2458/14/545/prepub

## Supplementary Material

Additional file 1 PRISMA Guideline checklist. Click here for file

Additional file 2 Additional characteristics of included review studies, grouped by ethnicity. Click here for file
