# Supplementary material for: Evidence for low high-density lipoprotein cholesterol levels in Australian indigenous peoples: a systematic review
Source: BMC Public Health. 2014 Jun 2;14:545. doi: 10.1186/1471-2458-14-545 (PMC4067101; doi:10.1186/1471-2458-14-545)
Supplement: Additional file 2 — Additional characteristics of included review studies, grouped by ethnicity. [file 1471-2458-14-545-S2.pdf]

Additional File 2: Additional characteristics of included review studies, grouped by ethnicity

Reference<br>Study date	Population type<br>Number (% female)<br>Age (years)<br>Response rate	Population health	% with HDL data	Setting	HDL assay type	HDL-C (mean ± SD) mmol/L	F:M HDL-C ratio	Study quality assessment
Indigenous populations								
[35]<br><br>Diabetes and related disorders in urban Indigenous people in the Darwin region (DRUID) study<br><br>2003-2005	84%AA, 6% TSI, 11% both<br><br>861 (68% F)<br>15-34 yrs: 394 (46%)<br>>35 yrs: 467 (54%)<br><br>14% of target population	In participants >35 yrs:<br>DM:<br>M: 23.3% F: 25.2%<br>CS:<br>M: 45.5% F: 42.9%	100%	Metropolitan Darwin, NT	Hitachi 917Enzymatic (after shipment to clinical trials medical laboratory in SA)	15-34 yrs:<br>M: 1.11 ± 0.30<br>F : 1.19 ± 0.33<br>>35 yrs:<br>M : 1.01 ± 0.29<br>F : 1.18 ± 0.34	15-34 yrs:<br>1.07<br>>35 yrs:<br>1.17	HIGH
[33]<br><br>KANYINI Audit study<br><br>2007-08	90% AA9%TSI, 1% both<br><br>1165 (59% F)<br><br>41.1 yrs (95% CI, 40.3-42.0)<br><br>N/A	OB: M: 31% F: 47%<br>DM: M: 21% F: 22%<br>CS: M: 61% F: 29%<br>29% patients aged ≥30yrs CVD high risk [1]	48%	8 health services in NSW, QLD, central Australia[2] in diverse settings	No standardization of methods across different laboratories/regions	M:<br>1.10 (SEM 0.02)<br>F:<br>1.20 (SEM 0.02)	1.0	MODERATE
Aboriginal populations								
[37]<br><br>1986	122 (59%F)<br><br>Adults > 17 yrs of age<br><br>>95% participation	11.5% with DM, 7.4% IGT<br>Mean BMI: (15-34 yrs)<br>M: 20.4 ± 0.6 kg/m^2<br>F: 20.5 ± 0.9 kg/m^2<br>(>35 yrs)<br>M: 23.5 ± 1.2 kg/m^2<br>F: 22.7 ± 0.9 kg/m^2	100%	1 isolated community in northern Australia	Enzymatic hydrolysis after PEG	Mean ± SE:<br>15-34 yrs:<br>M: 1.38 ± 0.08<br>women: 1.24 ± 0.05<br>>35 yrs:<br>M: 1.15 ± 0.08<br>F: 1.23 ± 0.07	15-34 yrs:<br>0.90<br>>35 yrs:<br>1.07	HIGH

[38]<br><br>1987	353 (69%F)<br><br>Adults > 15 yrs<br><br>87% participation	DM: (>35 yrs)<br>29.6%; (15-34 yrs) 5.3%<br>Overweight/obese (≥25 kg/m^2): (>35 yrs) M 51% F: 75%<br>upper tertile of 2-hr insulin distribution had significantly lower HDL-C: 1.33 ± 0.48 vs. 1.20± 0.40 (P=0.04)	100%	1 community, with a long history of acculturation in central Australia	Enzymatic hydrolysis (Cobas Bio Centrifugal Analyser) after PEG	15-34 yrs:<br>M: 1.20 ± 0.36<br>F: 1.39 ± 0.49<br>>35 yrs:<br>M: 1.04 ± 0.23<br>F: 1.25 ± 0.35	15-34 yrs:<br>1.16<br>>35 yrs:<br>1.20	HIGH
[23]<br><br>1988	437 (57% F f)<br><br>Adults ≥ 15 yrs<br><br>80% participation	With DM: 9%<br>IGT: 18%<br>Mean BMI: (15-34 yrs)<br>M: 21.8 ± 3.3 kg/m^2<br>F: 22.2 ± 5.3 kg/m^2<br>(>35 yrs)<br>M: 23.9 ± 4.6 kg/m^2<br>F: 24.2 ± 5.0 kg/m^2	100%	1 isolated community in central NT	Isolation after PEG	15-34 yrs:<br>M: 0.83 ± 0.20<br>F: 0.97± 0.26<br>>35 yrs:<br>M: 0.83 ± 0.22<br>F: 0.94 ± 0.23	15-34 yrs:<br>1.17<br>>35 yrs:<br>1.13	HIGH
[36]<br><br>1992-95	687 (48%F)<br><br>M: 34 ± 16 yrs<br>F: 36 ± 15 yrs<br><br>>80% of adults	With DM: M: 11% F: 16%<br>CS: M: 53% F: 16%<br>Mean BMI : M: 23.2 ± 4.6 kg/m^2 F: 24.1 ± 5.7 kg/m^2<br>Mean WC : M: 86.9 ± 13.1 cm F: 91.2 ± 14 cm	100%	1 community in remote region, Tiwi Islands, NT	Not stated	M: 1.1 ± 0.2<br>F: 1.0 ± 0.2	0.91	MODERATE
[24]<br><br>1993-95	852 (57% F)<br><br>M: 34 ± 16 yrs<br>F: 36 ± 15 yrs<br><br>NR	With DM: M: 9.3% F: 16%<br>CS: M: 83.7% F: 71%<br>Mean BMI (95% CI]): M: 26 (25.4-26.7) kg/m^2<br>F: 27.5 (26.9-28.1) kg/m^2	100%	11 remote communities in Cape York, QLD and Torres Strait and central Australia, NT	Standard enzymatic techniques (Boehringher Mannheim reagents)	M: 0.83 (95% CI 0.81, 0.95)<br>F: 0.88 (95% CI 0.85, 0.90)	1.06	MODERATE
[9]<br><br>1994	51 (39% F)<br><br>All community (n=77): these data were restricted to age range >15 yrs<br><br>100%	Low HDL (NHFA guidelines): M: 67% F: 95%<br>OS: M: 11% F: 40%<br>Central obesity: M: 48% (WHR >0.9) F: 90% (WHR >0.8)<br>Faecal parasites were prevalent; chronic infections common.	100%	1 isolated community in Great Sandy Desert, WA	Automatic Analyser (Hitachi 747) with PEG	M: 0.87 (95% CI 0.79, 0.94)<br>F: 0.82 (95% CI 0.73, 0.90)	0.94	HIGH

[21]<br><br>1996	171 (55% F)<br><br>M: 38 yrs (95% CI, 34-42)<br>F: 37 yrs (34-41)<br><br>67% of eligible adults	MetS (NCEP-ATPIII)<br>M: 27% F: 51%<br>CRP (GM [95% CI): M: 4.1 (CI 3.2-5.2) F: 6.6 (CI 5.4-8.1)<br>HDL-C inversely correlated with CRP: r= -.161 and soluble E-selectin r= -.163 (P<0.05)	100%	1 remote community in north-western WA	Standard, automated, colorimetric methods using commercial reagents (Hitachi 704 Analyser)	M: 0.86 (95% CI 0.81, 0.91)<br>F: 0.88 (95% CI 0.84, 0.92)	1.09	HIGH
[8]<br><br>1999-2000	237 (56% F)<br><br>M: 38.5 (CI 28.9-48.7)<br>F: 35.8 (CI 28.8-46.0)<br><br>58% eligible adults	Mean BMI (95% CI]): M: 23.9 (22.4-24.8) kg/m^2 F: 23.7 (22.8-24.6) kg/m^2<br>DM: M: 25% F: 25%<br>CS:<br>M: 81% F: 60%<br>CRP (GM [95% CI]): M: 6.4 (5.4-7.6) mg/L F: 8.0 (6.9-9.2) mg/L<br>HDL-C inversely correlated with IgG and fibrinogen	100%	1 remote community, East Arnhem land, NT	Standard, automated analysers (no further details)	GM (95% CI):<br>M: 0.83 (CI 0.78, 0.87)<br>F: 0.82 (CI 0.78, 0.86)	0.99	MODERATE
[39]<br><br>1987-88	306 (58% F)<br><br>M: 29.9 (SE 1.2)<br>F: 34.1 (SE 1.2) (Guest, O'Dea et al. 1992)<br><br>90%	DM: M: 8.8% F: 7.2% (Guest, O'Dea et al. 1992)<br>CS: M: 66.9% F: 63.1% (Guest, O'Dea et al. 1992)	100%	1 community in regional VIC	Automated enzymatic colorimetric analysis with PEG using Boehringher Mannheim commercial kits.	M: 1.6 (SEM 0.1)<br>F:<br>1.5 (SEM <0.1)	0.94	MODERATE

[32]<br><br>1998-2001<br><br>'Well Person's Health Check' study	1641 (54% F)<br><br>M: 36.6 (CI 35.5-37.7)<br>F: 37.4 (CI 36.4-38.4)<br><br>44.5%	DM: M: 9.5% F: 12.9%<br>CS:<br>M: 68.8% F: 56.0%<br>OB: M: 16.8% F: 26.8%	100%	23 rural communities in far north QLD	Photometric enzyme endpoint assay with Cobas Integra 700/400	M: 1.19 (95% CI 1.17-1.22)<br>F: 1.16 (95% CI 1.14-1.18)	0.97	MODERATE
[34]<br><br>Study conducted in 2001-03<br><br>Perth Aboriginal Atherosclerosis Risk Study	602 (86% F)<br><br>M: 40 (31-48)<br>F: 40 (32-48)<br><br>Approximately 20% of adults aged 25-64, <5% for ages 18-24	DM: M: 25% F: 25%<br>CS:<br>M: 45% F: 44%<br>OB M: 37% F: 68%<br>History of CVD: M: 18% F: 14%	100%	Metropolitan Perth, WA	No information other than at 'Department of Clinical Biochemistry, Path Centre laboratories, WA'<br>(no further info in other PAARS publications)	Median (IQR):<br>M: 1.0 (0.9-1.2)<br>F: 1.2 (1.0-1.5)	1.2	MODERATE
[20]<br><br>Study conducted in 2001-03	379 (54% F)<br><br>M: 32 (CI 31-34)<br>F: 36 (CI 34-37)<br><br>NR	Mean BMI (95% CI]): M: 21.6 (20.8-22.4) kg/m^2 F: 23.2 (22.5-23.8) kg/m^2<br>DM: M: 11% F: 13%<br>MetS (NCEP): M: 18% F: 41%<br>CS:<br>M: 65% F: 54%<br>CRP (geometric mean [95% CI]): M: 3.42 (2.94, 3.97) mg/L F: 4.06 (3.53, 4.66) mg/L	100%	Isolated coastal community in north-east Arnhem Land, NT	Standard automated colorimetric method	GM (95% CI):<br>M: 1.00 (CI 0.96-1.04)<br>F: 0.91 (CI 0.88-0.95)	0.91	MODERATE

[22]	348 (54% F)<br><br>152 aged 15-24 yrs<br>87 aged 25-34 yrs<br>109 aged >35 yrs<br><br>Study conducted in 1987<br><br>M:-83%<br><br>F: 90-96%	OB: 22.8%<br>DM: 11.6%	100%	1 rural Aboriginal community, NT	Standard enzymatic techniques with PEG using Boehringer Mannheim commercial kits.	Means (95% CI)<br>15-24 yrs:<br>M: 1.17 (1.10-1.24)<br>F: 1.45 (1.33-1.57)<br>25-34 yrs:<br>M: 1.23 (1.10-1.36)<br>F: 1.25 (1.12-1.38)<br>>35 yrs:<br>M: 1.05 (0.96-1.14)<br>F: 1.18 (1.10-1.27)	15-24 yrs:<br>1.24<br>25-34 yrs:<br>1.02<br>>35 yrs:<br>1.12	HIGH
	331 (58% F)<br><br>123 aged 15-24 yrs<br>85 aged 25-34 yrs<br>123 aged >35 yrs<br><br>Study conducted in 1991<br><br>M: 57-86%<br><br>F: 76-96%	OB: 32.0%<br>DM: 18.6%	100%			15-24 yrs:<br>M: 0.88 (0.82-0.94)<br>F: 0.98 (0.91-1.05)<br>25-34 yrs:<br>M: 0.84 (0.77-0.91)<br>F: 0.81 (0.75-0.87)<br>>35 yrs:<br>M: 0.78 (0.72-0.81)<br>F: 0.84 (0.79-0.89)	15-24 yrs:<br>1.11<br>25-34 yrs:<br>0.96<br>>35 yrs:<br>1.08	MODERATE
	305 (58 % F)<br><br>97 aged 15-24 yrs<br>94 aged 25-34 yrs<br>114 aged >35 yrs<br><br>Study conducted in 1995<br><br>M 53-68%<br>F: 52-97%	OB: 37.0%<br>DM: 20.7%	100%			15-24 yrs:<br>M: 0.83 (0.76-0.90)<br>F: 0.88 (0.81-0.95)<br>25-34 yrs:<br>M: 0.79 (0.73-0.85)<br>F: 0.86 (0.81-0.92)<br>>35 yrs:<br>M: 0.76 (0.70-0.81)<br>F: 0.82 (0.78-0.86)	15-24 yrs:<br>1.06<br>25-34 yrs:<br>1.09<br>>35 yrs:<br>1.04	MODERATE

Torres Strait Islander populations								
[24]<br><br>1993-95	276 (54% F)<br>M: 36 ± 16 yrs<br>F: 38 ± 15 yrs<br><br>NR	DM: M: 17% F: 36%<br>CS: M: 57% F: 37%<br>Mean BMI (95% CI]): M: 29.6 (28.5-30.7) kg/m^2<br>F: 31.1 (30.1-32.2) kg/m^2	100%	11 remote communities in Cape York, QLD and Torres Strait and central Australia, NT	Standard enzymatic techniques (Boehringher Mannheim reagents)	M: 1.01(95% CI 0.96-1.06)<br>F: 1.00 (95% CI 0.95, 1.06)	0.99	MODERATE
[32]<br><br>1998-2001<br><br>'Well Person's Health Check' study	968 (49% F)<br><br>M: 37.5 (CI 36.2-38.9)<br>F: 38.3 (CI 36.9-39.8)<br><br>44.5%	DM: M: 16.7% F: 22.7%<br>CS: M: 55.5% F: 44.8%<br>OB: M: 43.4% F: 60.6%	100%	23 rural communities in far north QLD	Photometric enzyme endpoint assay with Cobas Integra 700/400	M: 1.13 (95% CI 1.10-1.15)<br>F: 1.11 (95% CI 1.09-1.13)	0.98	MODERATE

LEGEND:

Abbreviations: AA: Australian Aborigine; BMI: body mass index; F: female; m, male; DM: diabetes mellitus; IGT: impaired glucose tolerance; M: male: NCEP-ATPIII: National Cholesterol Education Program, Adult Treatment Panel III; MetS (NCEP-ATPIII): Metabolic Syndrome defined according to NCEP-ATPIII guidelines; PEG: polyethylene glycol precipitation; TSI: Torres Strait Islander; WC: waist circumference; CS, current smokers; OB, obesity (body mass index >30 kg/m^2); GM, geometric mean; NSW, New South Wales; QLD, Queensland; NT, Northern Territory; VIC, Victoria; WA, Western Australia
[1] by 2004 NHFA adjusted Framingham equation
[2]Seven Aboriginal Community Controlled Health Organization health services, one state government Indigenous health service
